# Association of Pulp Stones With Single Nucleotide Polymorphisms of Mineralization‐Related Genes in Orthodontically Treated Patients: A Cross‐Sectional Study

**DOI:** 10.1155/ijod/1623851

**Published:** 2026-08-06

**Authors:** Daniel Hemming, Bianca Marques de Mattos de Araujo, Christian Kirschneck, Rafaela Scariot, Camila Paiva Perin, Manoel D. Sousa-Neto, Gabriela Fonseca-Souza, Natanael Henrique Ribeiro Mattos, Michelle Nascimento Meger, Flares Baratto-Filho, Erika Calvano Küchler

**Affiliations:** ^1^ School of Dentistry, Tuiuti University of Paraná, Curitiba, Paraná, Brazil, utp.edu.br; ^2^ Department of Orthodontics, Medical Faculty, University Hospital Bonn, Bonn, Germany, uni-bonn.de; ^3^ Department of Stomatology, Federal University of Parana, Curitiba, Paraná, Brazil, ufpr.br; ^4^ Department of Restorative Dentistry, University of São Paulo, Ribeirão Preto, São Paulo, Brazil, usp.br; ^5^ School of Dentistry, University of the Joinville Region – Univille, Joinville, Santa Catarina, Brazil

## Abstract

**Aims:**

The current evidence suggests that the formation of pulp stones (PS) has a genetic background. Therefore, the present study aimed to evaluate the association between PS in orthodontically treated patients and single nucleotide polymorphisms (SNPs) in the mineralization‐related genes (*bone morphogenetic protein 2* [*BMP2]*, *BMP4*, *SMAD6*, and *runt-related transcription factor 2* [*RUNX2]*).

**Materials and Methods:**

This cross‐sectional study analyzed genomic DNA and panoramic radiographs from patients with a history of orthodontic treatment. Panoramic radiographs were analyzed and PS were identified as well‐ or poorly defined radiopaque opacities located within the pulp chamber of the maxillary and mandibular first and second permanent molars. Genomic DNA obtained from all participants was analyzed. The genetic analysis focused on seven specific SNPs located in genes involved in mineralization pathways: *BMP2* (rs235768 and rs1005464), *BMP4* (rs17563), *SMAD6* (rs3934908 and rs2119261), and *RUNX2* (rs59983488 and rs1200425). Comparisons of allele and genotype (codominant, dominant, and recessive models) distributions between patients with and without PS were performed using the chi‐square test. Logistic regression analyses were performed to control for potential confounders such as age and sex for the SNPs located in the *RUNX2* gene. One‐way ANOVA was applied to evaluate differences in the mean number of teeth with PS across genotypes. The significance level was established at *α* = 0.05.

**Results:**

The final sample comprised 235 patients: 120 presented with PS, and 115 served as controls. For the SNP rs59983488 in *RUNX2*, a statistical association was observed for genotype distribution in the dominant model (*p* = 0.033) and for allele distribution (*p* = 0.022). For the SNP rs1200425 in *RUNX2*, a statistical association was observed for genotype distribution in the recessive model (*p* = 0.046; odds ratio [OR] = 2.0, confidence interval [CI] 95% 1.0–3.8). No statistically significant association was observed in the logistic regression analysis.

**Conclusion:**

Our study suggests that RUNX2 could have a small effect on the susceptibility of orthodontic patients to develop PS.

## 1. Introduction

Pulp calcification is considered an abnormal tissue response of the dental pulp to a variety of stimuli, including chronic inflammation, trauma, and restorative procedures. These calcifications, known as pulp stones (PS), can impair endodontic treatment by altering access cavity morphology, limiting canal instrumentation, and increasing the risk of procedural complications [1–3]. According to a systematic review and meta‐analysis, the overall prevalence of PS was estimated at 36.5% for individuals and 9.57% for the examined teeth, demonstrating that this is a relatively common phenomenon in the general population [4]. However, despite their frequency, the underlying biological mechanisms responsible for the formation of PS remain incompletely understood. Various etiological factors have been proposed for pulp calcification, including aging, trauma, orthodontic treatment, and genetic conditions [5–8]. Orthodontic tooth movements trigger a complex series of tissue reactions affecting the periodontal tissue and pulpal structures and might increase the risk of PS [9, 10].

The biological process underlying pulp calcification involves the differentiation of odontoblast‐like cells and the deposition of a mineralized matrix, which may be modulated by proinflammatory signals and molecular regulators. Some genes are candidates for pulp calcification due to their role in dental tissue development and calcification. The *bone morphogenetic protein* (*BMP*) family plays an essential role in dentin‐pulp complex homeostasis and repair. Specifically, *BMP2* and *BMP4* genes have been demonstrated to promote the differentiation of the odontoblast: dentin formation is upregulated following pulp injury or inflammation [11, 12]. *Runt-related transcription factor 2* (*RUNX2*), a key transcription factor involved in osteoblast and odontoblast differentiation, also plays an essential role in bone formation and dentinogenesis, which is the formation of dentin [13]. Acting downstream of *BMP* signaling, *RUNX2* regulates genes critical for mineralized tissue development and works in cooperation with *BMP2* and *BMP4* [13]. On the other hand, *SMAD6* (*SMAD family member 6*) acts as a negative regulator of *BMP* signaling. It prevents the phosphorylation of receptor‐regulated *SMADs*, thus limiting the mineralization activity [14].

Single nucleotide polymorphisms (SNPs) in *BMP*‐related genes and in *RUNX2* may influence the activity of mineralization pathways and thus affect susceptibility to calcification in the dental pulp. Previous studies have demonstrated associations between SNPs and pathological calcifications in other tissues, such as kidney stones, and have suggested a potential link between pulp and systemic mineralization processes [15]. Moreover, preliminary studies investigating SNPs in candidate genes [7, 8, 16] indicate that genetic variants may modulate the risk of developing PS [8, 13].

Research on the association between SNPs and PS remains limited, and only a few studies have evaluated candidate genes associated with PS so far [7, 8, 16, 17]. Investigating the genetic background of PS formation in orthodontically treated patients is highly important [7, 8]. We hypothesize that genetic variants in key genes are associated with patients’ susceptibility to altered mineral deposition in the dental pulp. In this context, the present study aimed to evaluate the association between PS in molars of orthodontically treated patients and SNPs in the mineralization‐related genes (*BMP2*, *BMP4*, *SMAD6*, and *RUNX2*).

## 2. Materials and Methods

### 2.1. Sample

This research project was approved by the local Ethics Committee under Protocol Number CAAE 8084631.0000.0093 and adhered to the ethical principles of the Declaration of Helsinki. A cross‐sectional study was conducted in Brazil using data collected between 2017 and 2020. The study analyzed pre‐existing genotype data from genomic DNA extracted from saliva and panoramic radiographs from dental patients. The saliva collection was performed during the dental appointment and was previously described [18]. No additional radiographs were performed for the current study. Participants were consecutively selected based on their availability, ensuring that all individuals who met the inclusion criteria during the study period were considered. The sample size calculation was based on genotype and PS frequencies, indicating that a minimum of 81 individuals per group was required to detect a frequency difference of 0.2 (degrees of freedom = 1, *α* = 0.05, and *β* = 0.80).

The investigation focused on evaluating the occurrence of pathological pulp calcifications, defined by the presence of PS. For this purpose, panoramic radiographs were collected from all patients and subsequently analyzed. PS were identified as well‐ or poorly defined radiopaque opacities located within the pulp chamber of the maxillary and mandibular first and second permanent molars. Each radiograph was carefully examined to determine the presence or absence of PS in every molar. This systematic approach ensured that the diagnosis of pulp calcification was standardized and reproducible across the entire sample.

Participants were eligible for inclusion if they presented complete root formation (apex closure) in the first and second molars, had at least one permanent molar present in the oral cavity, and provided panoramic radiographs of adequate diagnostic quality. Exclusion criteria comprised radiographs with poor image quality hindering diagnostic interpretation, syndromic conditions, the presence of cleft lip and/or palate, teeth with extensive coronal destruction, patients using orthodontic appliances at the time of imaging, and teeth with extensive restorations preventing visualization of the pulp chamber.

### 2.2. Radiographic Assessment and Calibration

All panoramic radiographs were digitalized and subsequently analyzed by a specialist in endodontics, who was responsible for diagnosing the presence of PS throughout the study. Panoramic radiographs were used because they were the only available records, thereby avoiding unnecessary radiation exposure to the patients. To minimize diagnostic variability and ensure methodological rigor, the examiner underwent a calibration process under the supervision of an experienced endodontist. The examiner was blinded to the genetic data.

The calibration procedure involved the independent evaluation of a preliminary set of 10 panoramic radiographs not included in the study sample. Following this step, consensus discussion was carried out to resolve discrepancies and establish uniform diagnostic criteria. This process guaranteed that the examiner was trained to recognize both subtle and well‐defined cases of PS. Then, to evaluate the reliability of PS diagnosis, a kappa statistic calculation was performed. The resulting kappa value was greater than 0.9, indicating excellent intraexaminer agreement. After calibration, the evaluation of the complete dataset was performed exclusively by the same examiner, which eliminated the possibility of inter‐examiner variability and strengthened the internal consistency of the diagnostic procedure.

### 2.3. DNA Extraction and Genotyping

Genomic DNA obtained from buccal epithelial cells of all participants and stored was used for genotyping analysis. The extraction was performed as previously described. Briefly, genomic DNA was isolated from the sample that was collected from epithelial cells through a 3% glucose mouthwash with a buccal swab [18]. The concentration and purity of the isolated genomic DNA were measured using a NanoDrop 2000 spectrophotometer (Thermo Fisher Scientific, Waltham, MA, USA). The samples were stored at −20°C in the laboratory until genotyping.

The genetic analysis focused on seven specific SNPs located in genes involved in mineralization pathways: *BMP2* (SNPs rs235768 and rs1005464), *BMP4* (SNP rs17563), *SMAD6* (SNPs rs3934908 and rs2119261), and *RUNX2* (SNPs rs59983488 and rs1200425). The SNPs and gene characteristics are presented in Table 1.

**Table 1:** Genes and SNPs characteristics.

SNP	Gene	Gene name	Functional consequence^a^	Clinical significance	Chromosomes	Base change	Genotyping call rate	HWE *χ* ^2^
rs235768	*BMP2*	*Bone morphogenetic protein 2*	Synonymous variant, missense variant, coding sequence variant	Benign‐likely	20p12	A > T	98.72	0.521
rs1005464			Intron variant	Not reported		A > G	99.57	0.412

rs17563	*BMP4*	*Bone morphogenetic protein 4*	Missense variant, coding sequence variant	Benign‐likely	14q22	G > A	99.15	1.847

rs3934908	*SMAD6*	*SMAD family member 6*	Genic downstream transcript variant, intron variant	Not reported	15q22	T > C	97.45	0.012
rs2119261			Genic downstream transcript variant, intron variant	Not reported		T > C	97.45	0.176

rs59983488	*RUNX2*	*Runt-related transcription factor 2*	Upstream transcript variant, intron variant	Not reported	6p21	T > G	97.02	3.349
rs1200425			Intron variant, genic downstream transcript variant	Not reported		A > G	98.72	1.475

^a^ClinVar.

Genotyping was made using TaqMan SNP Genotyping Assays (Applied Biosystems, Foster City, CA, USA) in real‐time PCR reactions. Each reaction was conducted in a 96‐well plate format. Genotyping was performed by allelic discrimination real‐time PCR using the TaqMan assay in a StepOnePlus thermocycler (Thermo Fisher). In each well of the plate, 1 µL (4 ng) of DNA was pipetted plus 2 µL of the reaction containing 1.5 µL of Master Mix, 0.037 µL of 40x SNP, and 0.462 µL of sterilized water, finally covered with an optical adhesive film (MicroAmp Optical Adhesive Film, Thermo Fisher Scientific, MA, USA). The thermal cycling condition used was a standard protocol, including an initial denaturation step at 95°C for 5 min. This was followed by 40 cycles of a two‐step program: denaturation at 92°C for 15 s and a combined annealing and extension at 60°C for 60 s. Fluorescence data, specifically from the FAM and VIC reporter dyes, were collected.

The overall genotyping success rate across all SNPs and samples was 98.3% (Table 1), indicating high reliability of the genotyping data.

### 2.4. Statistical Analysis

The Hardy–Weinberg equilibrium (HWE) was tested to ensure the reliability of the genotyping data (Table 1). Descriptive statistics were used to characterize the study population in terms of age, sex, and the presence of PS. Allele and genotype frequencies were calculated for all SNPs investigated. Comparisons of allele and genotype (codominant, dominant, and recessive models) distributions between patients with and without PS were performed using the chi‐square test.

Logistic regression analyses were performed to control for potential confounders such as age and sex for the SNPs located in the *RUNX2* gene (rs59983488 and rs1200425) that presented nominative *p*‐values lower than 0.05.

To further explore possible associations, Student’s *t* test was used to compare mean age between groups, while one‐way ANOVA was applied to evaluate differences in the mean number of teeth with PS across genotypes.

Odds ratios (ORs) and 95% confidence intervals (95% CIs) were calculated to quantify the strength of associations. All statistical analyses were performed using SPSS, with the significance level established at *α* = 0.05.

## 3. Results

The final sample comprised 235 patients, of whom 120 (51.05%) presented PS and 115 (48.95%) served as controls without PS. In the control group, the mean age was 26.0 (standard deviation 8.76), and in the PS group, it was 27.9 (standard deviation 7.68). Regarding sex distribution, 63 females and 52 males were included in the control group, while 80 females and 40 males presented PS and were the case group. Statistical analyses showed that sex was not statistically significantly associated with PS (*p* = 0.088). Age distribution was also not statistically different between groups (*p* = 0.087) (Table 2).

**Table 2:** Sample characteristics.

Variables	Control group (*n* = 115)	Pulp stones group (*n* = 120)	*p*‐Values
Sex, *N* (%)			
Female	63 (54.8%)	80 (66.6%)	0.088^a^
Male	52 (45.2%)	40 (33.3%)	
Age (in years)			
Minimum–maximum	17–47	17–38	0.087^b^
Mean (standard deviation)	26.03 (SD = 8.76)	27.88 (SD = 7.68)	

Abbreviation: SD, standard deviation.

^a^Chi‐square with Yates correction was used.

^b^ *t*‐Test was used.

Genotype distributions of all investigated SNPs were consistent with the HWE.

Table 3 shows genotype and allele distributions between the control group and PS group. For the SNP rs59983488 in *RUNX2*, a statistical association was observed for genotype distribution in the dominant model (*p* = 0.033; OR = 1.8, CI 95% 1.1–3.1) and for allele distribution (*p* = 0.022; OR = 1.7, CI 95% 1.1–2.7), in which, carrying the T allele increases the chance of having PS. For the SNP rs1200425 in *RUNX2*, a statistical association was observed for genotype distribution in the recessive model (*p* = 0.046; OR = 2.0, CI 95% 1.0–3.8) in which carrying 2 A alleles increases the chance of having PS.

**Table 3:** Frequency distribution of genotypes among the groups.

Gene	SNP	Control group			Pulp stone group			*p*‐Values codominant model	*p*‐Values dominant model	*p*‐Values recessive model	*p*‐Values allele
		Genotypes *N*			Genotypes *N*						
*BMP2*	rs235768	AA	AT	TT	AA	AT	TT	0.983	0.982	0.923	0.933
		14	46	53	14	51	54				
	rs1005464	AA	AG	GG	AA	AG	GG	0.969	0.954	0.911	0.942
		7	35	72	7	38	75				

*BMP4*	rs17563	GG	AG	AA	GG	AG	AA	0.503	0.304	0.370	0.234
		29	55	30	24	57	38				

*SMAD6*	rs3934908	TT	CT	CC	TT	CT	CC	0.409	0.455	0.421	0.399
		23	56	31	27	66	26				
	rs2119261	TT	CT	CC	TT	CT	CC	0.370	0.409	0.894	0.957
		25	48	38	21	63	34				

*RUNX2*	rs59983488	TT	GT	GG	TT	GT	GG	0.061	**0.033**	0.129	**0.022**
		6	43	62	2	33	82				
	rs1200425	AA	AG	GG	AA	AG	GG	0.068	0.529	**0.046**	0.383
		27	51	34	16	69	35				

*Note:* Chi‐square test was used. Total genotype counts may not equal the total sample size due to genotyping call rates. Bold values indicate statistical significance difference.

Table 4 shows the logistic regression analysis for the SNPs in *RUNX2*. A statistically significance association was not observed for genotypes with PS (*p*  > 0.05).

**Table 4:** Logistic regression analysis of the SNPs in *RUNX2* and PS phenotype.

Variables	Estimate	95% CI	*p*‐Values
*rs59983488*			

Age	−0.01	−0.03 to 0.01	0.319
Sex	0.55	0.01–1.10	0.074
GG vs. TT	0.77	−0.69 to 2.39	0.309
GT vs. TT	0.23	−1.26 to 1.88	0.767

*rs1200425*			

Age	0.99	0.97–1.01	0.294
Sex	1.59	0.93–2.74	0.091
AG vs. GG	1.21	0.66–2.22	0.526
AA vs. GG	0.53	0.24–1.16	0.119

Abbreviation: CI, confidence interval.

Table 5 shows the mean number of teeth with PS per genotype. A statistical association was not observed with PS (*p* > 0.05).

**Table 5:** Mean number of teeth with PS per genotype.

SNP	Genotype	Mean	SD	*p*‐Values		
				Codominant model^a^	Dominant model^b^	Recessive model^b^
rs235768	AA	1.929	2.210	0.759	0.785	0.559
	AT	2.113	2.445			
	TT	2.299	2.816			

rs1005464	AA	2.000	2.828	0.878	0.873	0.811
	AG	2.288	2.674			
	GG	2.122	2.529			

rs17563	GG	2.515	2.862	0.347	0.702	0.147
	AG	1.964	2.274			
	AA	2.019	2.671			

rs3934908	TT	2.100	2.468	0.584	0.522	0.673
	CT	2.328	2.608			
	CC	1.912	2.537			

rs2119261	TT	1.913	2.606	0.211	0.277	0.208
	CT	2.468	2.710			
	CC	1.847	2.250			

rs59983488	TT	1.750	3.412	0.278	0.109	0.345
	GT	1.789	2.241			
	GG	2.340	2.608			

rs1200425	AA	1.488	2.208	0.111	0.921	0.061
	AG	2.450	2.589			
	GG	2.159	2.747			

Abbreviation: SD, standard deviation.

^a^One‐way ANOVA was used.

^b^ *t*‐Test was used.

## 4. Discussion

The identification of SNPs serving as genetic markers for PS susceptibility carries several clinical implications for the orthodontic management of these patients. From a diagnostic perspective, understanding a patient’s genetic predisposition could aid clinicians in interpreting radiopaque findings on initial panoramic radiographs. Regarding risk stratification, patients harboring the identified risk alleles could be classified as a “high‐susceptibility” group. This is clinically significant because the presence of PS can complicate endodontic access, and some evidence suggests a shared pathway between pulp calcification and systemic conditions [1, 5, 6]. Consequently, for these high‐risk individuals, treatment planning might be modified to include more conservative force application protocols and more frequent radiographic monitoring. By integrating genetic risk profiles with routine clinical findings, orthodontists can transition toward a more personalized approach, such as anticipating calcific changes. Therefore, the present study investigated the association between PS and SNPs in *BMP2*, *BMP4*, *SMAD6*, and *RUNX2* in a sample of Brazilian patients that had orthodontic treatment. Although the genes selected for analysis participate in biological pathways relevant for mineralization, odontoblast differentiation, and dentinogenesis, the present findings revealed only a weak significance for SNPs in *RUNX2* but not for *BMP2*, *BMP4*, and *SMAD6*.

*BMP* signaling plays a central role in tooth development, pulp homeostasis, and reparative dentin formation, particularly through *BMP2* and *BMP4*, which regulate early odontoblast differentiation [1, 4]. Dysregulation of *BMP* signaling may theoretically predispose to ectopic mineral deposition, while *SMAD6* acts as an intracellular inhibitory molecule capable of dampening BMP‐driven mineralization processes [12]. A previous study from Thuller et al. [8] also investigated SNPs in *BMP2*, *BMP4*, and *SMAD6* associations with PS. In this previous study, an association was not observed for *BMPs*, but a borderline significance was observed for SNPs in *SMAD6*. Thus, we hypothesized that risk‐associated variants in SMAD6 may impair its ability to inhibit BMP signaling, thereby leading to uncontrolled mineralization and increased susceptibility to PS formation. The discrepancy between the previous study and ours may rely on population differences, given that our study specifically focused on patients undergoing orthodontic treatment, unlike Thuller et al. [8]. It is also possible that type I or type II error occurred, leading to a false positive or false negative result. The finding presented here reinforces the idea that PS is a multifactorial trait influenced by both environmental stimuli and numerous genetic variants with small effect sizes.

*RUNX2* is a master transcription factor in osteogenic and odontogenic lineage commitment and acts downstream of *BMP* signaling, regulating the expression of genes required for dentin and bone matrix formation [13]. The two studied SNPs in *RUNX2* were associated with PS in our current study only in univariate analysis. In the logistic regression analysis with age and gender as co‐variants, this statistical difference was not observed. We performed a multivariate analysis because age and sex showed a trend (*p*  < 0.10), with females and older patients presenting a higher frequency of PS. The fact that statistical differences were not sustained after logistic regression adjustment for age and sex could indicate that demographic variables may confound unadjusted associations—a phenomenon also described by other authors studying pulp mineralization [17, 19].

Previous studies also observed that SNPs, such as rs1017799, rs3806557, and rs10177996, could be involved in higher susceptibility of orthodontic patients to developing pulp calcification. Ramirez et al. [7] identified associations between pulp calcifications (including PS) and SNPs in the genes encoding wnt family members (*WNT6* and *WNT10A*), suggesting the involvement of *WNT*/*BMP* crosstalk in pulp mineralization. Additional studies have explored variants in *PTH* [15], growth factors [16], and other mineralization‐related genes, producing heterogeneous results and inconsistent replications across populations. However, the current study and previous studies present some methodological variations. Two previous studies did not focus on orthodontic patients [8, 15], while two others targeted orthodontic patients but evaluated all types of pulp calcification rather than isolating PS [7, 16]. Together, these findings highlight that PS formation may not be governed by single‐gene effects but rather by the combined influence of multiple pathways, including *BMP*, *WNT*, and inflammatory regulators.

The candidate genes and SNPs were selected based on direct and indirect evidence in the literature. *BMP2* is known to be required for odontoblast differentiation and the formation of the vascular bed in the dental pulp [20]. While no direct link between these specific SNPs (rs235768 and rs1005464) and PS was made, the biological plausibility remains. BMP4 is also crucial for tooth tissue formation. Its involvement in the BMP/SMAD cascade means that any SNP in *BMP4* that affects its binding or secretion could theoretically shift the balance of the dental pulp toward a pro‐calcifying environment. SMAD6 acts as an intracellular inhibitor of BMP signaling. It binds to type‐I BMP receptors to prevent the phosphorylation of R‐SMADs, effectively “turning off” the signal for mineralization. Research indicates that the rs2119261 and the haplotype (rs2119261 and rs3934908) were significantly associated with an increased risk of PS under a recessive model [8]. Plausibly, these SNPs may reduce the inhibitory efficiency of the SMAD6 protein. *RUNX2* is known as the “master switch” for mineralization. It is essential for both osteoblast and odontoblast differentiation. RUNX2 regulates the expression of major bone‐ and tooth‐related proteins. Although a direct association of rs59983488 and rs1200425 with PS was not significant in a recent study [8], our hypothesis was that RUNX2 remains biologically central and these SNPs could be involved in the risk to develop PS.

Another important aspect that should be discussed is the functional effects of the analyzed SNPs. The SNPs rs235768, rs1005464, rs17563, rs3934908, rs2119261, rs59983488, and rs1200425 exhibit diverse functional mechanisms (https://www.ncbi.nlm.nih.gov/snp/) [21] that can influence gene expression and protein activity. Specifically, rs235768 (in the *BMP2* gene) and rs17563 (in the *BMP4* gene) are missense variants that result in amino acid substitutions—arginine to serine and valine to alanine, respectively—which can alter protein expression, receptor binding affinity, mRNA stability, and downstream signaling pathways. rs59983488 is an upstream variant located in the 5^′^ untranslated region (UTR) of the *RUNX2* gene, a position that allows it to directly impact gene expression levels. The remaining polymorphisms—rs1005464 (*BMP2*), rs1200425 (*RUNX2*), and both rs3934908 and rs2119261 (*SMAD6*)—are classified as intronic variants situated in noncoding regions. While the precise molecular pathways for some of these noncoding variants remain to be fully elucidated, intronic polymorphisms such as rs1005464 and rs1200425 have the potential to induce aberrant mRNA splicing or otherwise affect the rate, efficiency, and accuracy of gene transcription.

The exact pathophysiology involved in PS formation is not well understood [2, 22, 23]. Interestingly, various systemic conditions [1, 6, 24–30], mainly metabolic diseases such as diabetes and cardiovascular diseases such as atherosclerosis, have been associated with PS. A study using histopathological sections observed that PS was associated with type II diabetes and hypertension [31]. In another study, PS showed a heterogeneous structure and chemical composition, and some elements showed concentrations higher in PS compared with healthy dentin [32]. These corroborate that the molecular mechanism underlying PS formation is highly complex.

Orthodontic forces promoted alterations in the dental pulp, producing clinical and radiographic findings [33]. Because our study exclusively sampled individuals with a history of orthodontic therapy, the findings regarding the genetic susceptibility to PS can only be generalized to the population of orthodontically treated patients and not to the broader, untreated sample.

The present study incorporates important methodological strengths that reinforce the reliability of its findings. The inclusion and exclusion criteria were rigorously defined, allowing the selection of individuals with complete root formation and molars free of extensive coronal destruction or large restorations. This ensured a radiographically stable and comparable pulp chamber morphology among all participants. Nevertheless, some limitations must be acknowledged. As a candidate‐gene study, the sample size may have been insufficient to detect SNPs with small effect sizes, which are typical of mineralization‐related traits. Because the analyzed variants are largely intronic or missense, the study is limited by a lack of direct functional or regulatory evidence, though these SNPs may potentially serve as proxies through linkage disequilibrium. Environmental and clinical factors known to influence pulp mineralization, including type of orthodontic biomechanics, subclinical inflammation, trauma, and systemic mineral metabolism, were not assessed and may act as relevant confounders [5]. Another important limitation is the absence of correction for multiple comparisons, which could lead to the possibility of false‐positive findings. Finally, panoramic radiographs, although advantageous for standardized evaluation across all molars, may underestimate subtle or early‐stage calcifications detectable only through higher‐resolution modalities, potentially resulting in conservative phenotype classification. To build upon this methodology, future research could integrate genetic analysis by utilizing DNA samples from patients who already have available cone‐beam computed tomography (CBCT) scans, which are the best exams to detect PS [34, 35], allowing for a comprehensive investigation into the factors underlying PS formation without exposing subjects to additional radiation.

In conclusion, our study suggests that the SNPs rs59983488 and rs1200425 in *RUNX2* could have a small effect on the susceptibility of orthodontically treated patients developing PS; however, this association was not observed in the multivariate analysis. SNPs in BMP2, BMP4, and SMAD6 were not associated with PS. Future studies should be performed in other populations investigating a larger number of SNPs in candidate genes.

## Author Contributions

Conceptualization: Erika Calvano Küchler, Manoel D. Sousa‐Neto, Flares Baratto‐Filho, and Natanael Henrique Ribeiro Mattos. Methodology: Camila Paiva Perin, Daniel Hemming, Bianca Marques de Mattos de Araujo, Michelle Nascimento Meger, Rafaela Scariot, and Camila Paiva Perin. Software: Erika Calvano Küchler and Manoel D. Sousa‐Neto. Validation: Christian Kirschneck, Gabriela Fonseca‐Souza, and Michelle Nascimento Meger. Formal analysis: Erika Calvano Küchler, Rafaela Scariot, Gabriela Fonseca‐Souza, Michelle Nascimento Meger, Gabriela Fonseca‐Souza, and Daniel Hemming. Investigation: Michelle Nascimento Meger and Daniel Hemming. Resources: Erika Calvano Küchler, Rafaela Scariot, Christian Kirschneck, and Flares Baratto‐Filho. Data curation: Manoel D. Sousa‐Neto and Natanael Henrique Ribeiro Mattos. Writing – original draft: Daniel Hemming and Erika Calvano Küchler. Visualization: Daniel Hemming, Michelle Nascimento Meger, Christian Kirschneck, Flares Baratto‐Filho, and Bianca Marques de Mattos de Araujo. Supervision: Bianca Marques de Mattos de Araujo, Flares Baratto‐Filho, Rafaela Scariot, and Erika Calvano Küchler. Project administration: Erika Calvano Küchler, Michelle Nascimento Meger, and Rafaela Scariot. Funding acquisition: Erika Calvano Küchler, Christian Kirschneck, and Rafaela Scariot. Writing – review and editing: Daniel Hemming, Michelle Nascimento Meger, Christian Kirschneck, Rafaela Scariot, Flares Baratto‐Filho, Bianca Marques de Mattos de Araujo, Manoel D. Sousa‐Neto, Natanael Henrique Ribeiro Mattos, and Camila Paiva Perin.

## Funding

No external funding was provided in regard with this study. The authors received no other institutional funding beyond their employment. Open Access funding enabled and organized by Projekt DEAL.

## Disclosure

All the authors have read and approved the final version of the manuscript. All the authors consent to the publication of this work.

## Ethics Statement

This study was approved by the Research Ethics Committee of the University, Curitiba, Paraná, Brazil (CAAE 80846317.8.0000.0093).

## Consent

All research participants were invited to participate in the study and those who agreed to participate read and signed the informed consent form.

## Conflicts of Interest

The authors declare no conflicts of interest.

## Data Availability

The data that support the findings of this study are available from the corresponding author upon reasonable request.
